# Endothelial Mechanosignaling: Does One Sensor Fit All?

**DOI:** 10.1089/ars.2015.6493

**Published:** 2016-09-01

**Authors:** Chris Givens, Ellie Tzima

**Affiliations:** ^1^ Department of Cell Biology and Physiology, University of North Carolina-Chapel Hill, Chapel Hill, North Carolina.; ^2^ Cardiovascular Medicine, Wellcome Trust Centre for Human Genetics, Oxford, United Kingdom.

## Abstract

***Significance:*** Forces are important in the cardiovascular system, acting as regulators of vascular physiology and pathology. Residing at the blood vessel interface, cells (endothelial cell, EC) are constantly exposed to vascular forces, including shear stress. Shear stress is the frictional force exerted by blood flow, and its patterns differ based on vessel geometry and type. These patterns range from uniform laminar flow to nonuniform disturbed flow. Although ECs sense and differentially respond to flow patterns unique to their microenvironment, the mechanisms underlying endothelial mechanosensing remain incompletely understood. ***Recent Advances:*** A large body of work suggests that ECs possess many mechanosensors that decorate their apical, junctional, and basal surfaces. These potential mechanosensors sense blood flow, translating physical force into biochemical signaling events. ***Critical Issues:*** Understanding the mechanisms by which proposed mechanosensors sense and respond to shear stress requires an integrative approach. It is also critical to understand the role of these mechanosensors not only during embryonic development but also in the different vascular beds in the adult. Possible cross talk and integration of mechanosensing *via* the various mechanosensors remain a challenge. ***Future Directions:*** Determination of the hierarchy of endothelial mechanosensors is critical for future work, as is determination of the extent to which mechanosensors work together to achieve force-dependent signaling. The role and primary sensors of shear stress during development also remain an open question. Finally, integrative approaches must be used to determine absolute mechanosensory function of potential mechanosensors. *Antioxid. Redox Signal.* 25, 373–388.

## Introduction

Mechanical forces influence every area of biology, from early development to adult physiology and pathology. During development, left–right asymmetry of the growing embryo, pruning of the immature vascular plexus, and renal morphogenesis are all regulated by mechanical forces (89, 103). Similarly, in the adult organism, several physiological processes are dependent on mechanical force sensing, including the senses of touch and hearing, as well as pulmonary surfactant production resulting from breathing. There is also a dark side to force sensing, as tumor metastasis and atherosclerosis are regulated by pathological forces and resultant mechanosignaling (103).

In the cardiovascular system, forces are critical determinants of vascular homeostasis and pathological processes. Vascular smooth muscle cells increase collagen production in response to stretch, which contributes to normal collagen synthesis and turnover, but can also lead to the development of atherosclerosis (82, 121). In addition, increases in cardiac load due to exercise or hypertension lead to extensive cardiac remodeling known as cardiac hypertrophy (37). Devastating conditions, such as aortic dissection, are fundamentally problems of mechanobiology and result when wall stress exceeds the strength of arterial walls (73). In addition, inflammatory flow patterns, including disturbed and low flow conditions, contribute to focal atherosclerotic plaque formation, which is the pathology behind debilitating cardiovascular events such as stroke and myocardial infarction (58). The past 25 years have yielded many insights into the mechanisms behind shear sensing in endothelial cells (ECs), including the identification of many putative endothelial shear stress sensors. This review focuses on the shear stress mechanosensors that have been identified in ECs and categorizes them based on their subcellular localization: luminal, junctional, or basal.

## Shear Stress and EC Responses

**FIG. 1.**
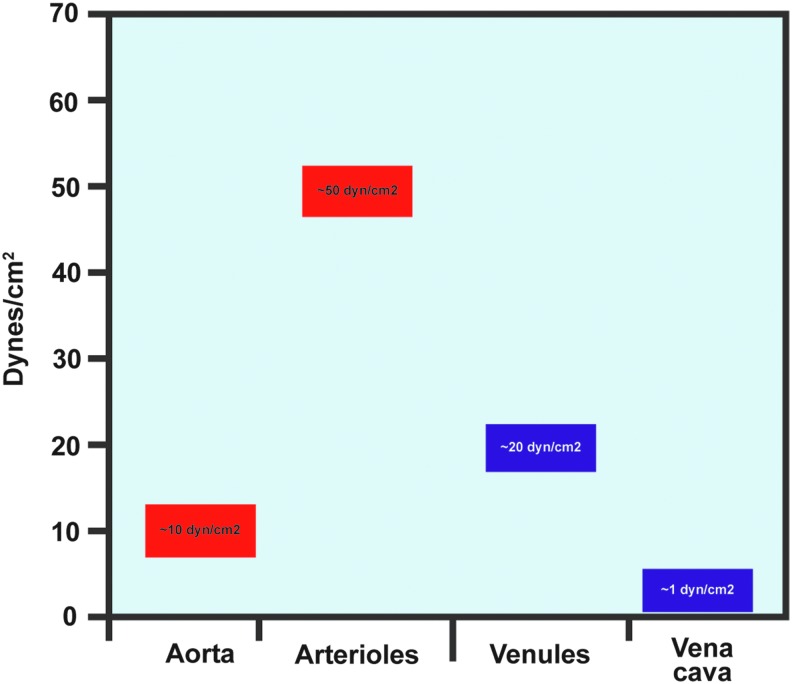
**Shear stress levels are variable throughout the vasculature.** Arterial shear stress levels are higher than venous levels, and larger vessels have lower shear than smaller vessels. To see this illustration in color, the reader is referred to the web version of this article at www.liebertpub.com/ars

The vasculature is constantly subjected to two main forces: circumferential stretch and fluid shear stress. The force of stretch, which results from the natural pulsatility of blood flow, is normal to the vessel wall (21, 134). Stretch can also arise as the result of chronic hypertension, causing thickening of arterial walls and decreasing responsiveness to vasodilatory stimuli (88). Stretch induces specific signaling pathways in the endothelium and vascular smooth muscle cells, leading to a complex suite of phenotypes and communication between the two cell types (4, 10, 88). Fluid shear stress is the frictional force felt by ECs as a result of blood flow parallel to the vessel wall (58). Shear stress is represented as a force per unit area; the most common units are dynes or Newtons. ECs throughout the vasculature experience a wide range of shear stresses and magnitudes (Fig. 1). Arterial shear stresses range from approximately 10 dyn/cm^2^ in the aorta to 50 dyn/cm^2^ in smaller arterioles (109). Shear stress in the venous circulation is lower, ranging from 20 dyn/cm^2^ in venules to 1 dyn/cm^2^ in the vena cava (85, 109).

**FIG. 2.**
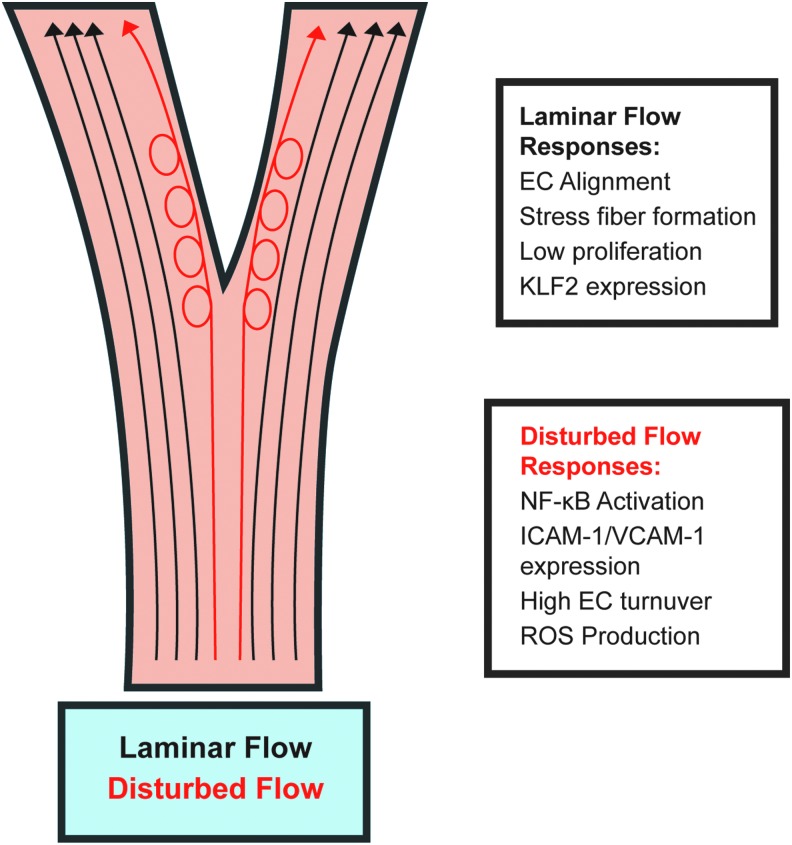
**Two main types of flow exist in the vasculature: laminar and disturbed flow.** Laminar flow occurs where vessel gemoetry is straight and uniform, whereas disturbed flow occurs where vessels bifurcate or curve highly. The different types of flow elicit different endothelial responses. Responses to laminar flow, termed “atheroprotective,” include EC alignment in the direction of flow, stress fiber formation, and KLF2 expression, which lead to anti-inflammatory gene expression. Disturbed flow responses, termed “atheroprone,” are more inflammatory and include NF-κB activation and associated transcription. In addition, ECs in areas of disturbed flow are more proliferative and produce more ROS than ECs in areas of laminar flow. EC, endothelial cell; ROS, reactive oxygen species. To see this illustration in color, the reader is referred to the web version of this article at www.liebertpub.com/ars

*In vivo*, blood flow exhibits differential patterns, which fall into two broad categories: laminar and disturbed flow, also known as atheroprotective and atheroprone flow, respectively (Fig. 2) (58). These flow patterns are determined by vessel geometry and give rise to vastly different gene expression profiles. The difference in flow patterns is sensed by endothelial mechanosensors, which transfer differential physical information into divergent biochemical signals. Laminar flow is characterized by high uniform flow and occurs mostly in straight areas of the vasculature, such as the descending aorta (17). ECs in areas of laminar flow align in the direction of flow, forming large stress fibers, and exhibit low cellular turnover. Anti-inflammatory genes are highly expressed, including the transcription factor KLF2 (17, 111). Conversely, disturbed flow is characterized by low and oscillatory flow patterns and is the main flow regimen in areas of the vasculature that are highly curved or bifurcated. ECs that experience disturbed flow do not align in the direction of flow and proliferate more rapidly (12, 17). Importantly, inflammatory gene expression is increased in ECs experiencing disturbed flow. Inflammatory adhesion molecules such as VCAM-1 are highly expressed, which can lead to recruitment of monocytes and formation of atherosclerotic plaques (90). In this manner, areas of disturbed flow are highly correlated with atherogenesis. In addition to systemic factors such as smoking or high blood cholesterol, EC inflammation caused by disturbed shear stress “primes” the endothelium for atherosclerotic plaque growth. A similar mechanism underlies the growth of collateral vessels after stenosis of the nutritive artery. Found throughout the body, collateral vessels are connectors between different arterial beds (43). Collaterals normally do not conduct blood, but upon stenosis of a major artery, blood flow markedly increases through the collaterals, increasing the shear stress experienced by ECs (15, 122, 123). This increase in shear stress leads to mechanotransduction and activation of signaling pathways that ultimately result in transient inflammation of the collateral ECs (122). Collateral ECs increase their proliferation and promote recruitment of immune cells (18, 123, 131). These processes guide collateral remodeling, and the collateral vessels eventually increase their caliber such that they conduct the amount of blood normally carried by the nutritive artery (122).

Endothelial responses to flow have been studied extensively *in vitro.* Early responses include K^+^ and Ca^2+^ influx, nitric oxide (NO) production, and reactive oxygen species production (70, 101, 102, 155). Soon thereafter, activation of MAP kinases, eNOS and Akt also occur. The discovery that shear stress activates integrins in ECs (76, 139, 140) opened up a new avenue of research on the importance of the identity of the extracellular matrix (ECM) in shear stress signaling (please see Review Forum article by Yurdagul and Orr). The current view is that the matrix and, as a result, the specific integrins that are binding to it serve as a “check-point” that determines which intracellular signaling responses will be activated and which will be inhibited. Examples include the activation of JNK when cells are plated on fibronectin and the activation of p38 MAP kinase when cells are on collagen (59, 87, 104). Similarly, the activation of p21-activated kinase and the inflammatory transcription factor NF-kB is also matrix specific, occurring specifically on fibronectin (104, 105)

Another important set of signaling molecules is also regulated by shear stress: members of the Rho family of GTPases are highly sensitive to both spatial and temporal regulation by shear stress (138). RhoA is transiently downregulated within 5 min of onset of shear stress, allowing breakdown and subsequent reformation of actin stress fibers, while Rac is transiently activated starting at 5 min and peaking at 30 min after onset of shear stress (139, 140). Interestingly, activation of Rho family members is subject to integrin control (138). Shear stress activates endothelial integrins, which form new connections with the ECM (140). This activity activates RhoA, which directs rearrangement of focal adhesions (27). In unstimulated ECs, the focal adhesion protein vinculin is evenly distributed in small puncta throughout the cell. However, after application of shear stress, vinculin aggregates into large focal adhesions at the upstream edge of ECs (50). Proper spatiotemporal activation of both Rho and Rac is required for alignment of ECs in the direction of flow (139, 140) as well as establishment of polarity in ECs (86). Cdc42 is also activated in response to shear (153) and its activation is required for polarization of the microtubule organizing center (MTOC) in ECs (142). Furthermore, EC migration speed increases when ECs are exposed to shear, an effect mediated by RhoA (126).

**FIG. 3.**
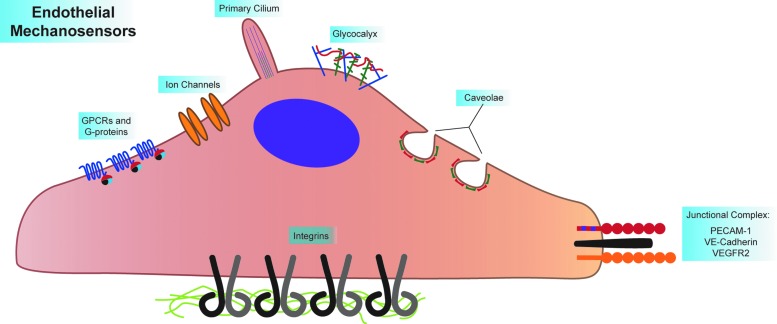
**Endothelial cells express many mechanosensors.** These sensors can be divided into luminal, junctional, and basal mechanosensors. Luminal mechanosensors include G-protein-coupled receptors (GPCRs, including the sphingosine 1-phosphate receptor 1, S1P_1_, and the bradykinin B2 receptor) and heterotrimeric G-proteins (namely, Gαq/11), ion channels (including TRPV4, TRPP2, TRPC1, Piezo1, and Piezo2), microtubule-based primary cilia (which associated with the ion channels PKD1 and PKD1), the glycocalyx (where Syndecan-1 and −4, as well as heparin sulfate glycosaminoglycans are involved in shear signaling), and protein-coated membrane pits called caveolae (given structure by Caveolin 1–3 and Cavin 1–3). The known junctional mechanosensors, PECAM-1, VE-Cadherin, and VEGFR2, form a mechanosensory complex that elicits many signaling pathways as a response to shear. The basal mechanosensors consist of the integrins, which sense ECM type and substrate stiffness, all while integrating signaling pathways originating from other mechanosensors. ECM, extracellular matrix; GPCR, G-protein-coupled receptors; PECAM-1, platelet endothelial cell adhesion molecule-1. To see this illustration in color, the reader is referred to the web version of this article at www.liebertpub.com/ars

Intermediate responses to shear include transcriptional activation of NF-kB target genes, such as ICAM-1 (104). In areas of laminar flow, shear signaling cascades culminate with downregulation of NF-kB, followed by atheroprotective transcription resulting from ERK5-dependent KLF2 expression (58, 111). At this time, ECs also align parallel to the direction of flow (140, 141). In areas of disturbed flow, however, NF-kB and other inflammatory signaling are sustained. Long-term EC inflammation leads to continuation of ICAM-1 and VCAM-1 expression, causing recruitment of monocytes to inflamed endothelium, beginning atherogenesis (17). In addition, increased fibronectin deposition occurs in areas of inflamed endothelium, enhancing and sustaining inflammation and atherogenesis in those areas (44, 53, 104). Extensive endothelial protein S-nitrosylation also occurs in response to shear stress (68). These proteins include heat shock proteins, as well as cytoskeletal elements such as tropomyosin and vimentin (71). The diverse suite of shear-induced endothelial signaling and phenotypes implies that ECs possess exquisite mechanosensitivity, which is indeed the case. ECs express many putative mechanosensors, the major groups of which are discussed below (Fig. 3).

## Apical Mechanosensors

### Primary cilia

Primary cilia are 3–5 μm long cellular protrusions provided structure by microtubular bundles. Embryonic evidence suggests a requirement for cilia in developing vasculature, where flow regimens are low. Developing zebrafish present with cranial hemorrhage after disruption of intraflagellar transport protein Ift81, while *Pkd2* EC-specific knockout causes hemorrhage in the cranium and back of developing mice (48, 60, 78, 111, 124).

EC primary cilia are primarily found in areas of the vasculature that experience low or disturbed flow. Their presence has been demonstrated in chicken embryonic endocardial and venous ECs (143). Cilia exist in embryonic mouse aorta and the inner curvature of adult mouse aorta, both low flow areas of the vasculature (101, 144). In addition, zebrafish arteries and veins exhibit cilia (52). The presence of cilia is known in primary chicken ECs (67), human umbilical vein ECs (HUVECs) (75), and embryonic mouse aortic ECs (101). Laminar shear causes rapid disassembly of cilia in ECs, and as such the contribution of cilia to long-term shear stress signaling remains unclear (75).

A body of research investigating cilia-dependent shear responses shows a role for cilia in early calcium signaling and nitric oxide (NO) production. To mediate shear-induced calcium and NO signaling, primary cilia partner with polycystin 1 and 2 (PKD1 and PKD2, expressed from the *Pkd1* and *Pkd2* genes). PKD1, a G-protein-coupled receptor (GPCR), constitutively activates Gi/o proteins. Physical association with PKD2, a Ca^2+^ channel, represses this constitutive activation (34). Using microfluorimetry, early shear-dependent calcium influx and NO production were examined *in vitro* using aortic ECs from *Pkd1*^null/null^ mice. Twenty seconds after the application of shear, high levels of calcium influx and NO production were observed in wild-type cells (101). However, early calcium influx and NO production were blocked in *Pkd1*^null/null^ ECs. In addition, *Pkd2* is required for shear-induced calcium influx and NO production. These early events are blocked by *Pkd2* knockdown in ECs (1). Interestingly, ECs isolated from an autosomal dominant polycystic kidney disease patient show heterogeneous expression of PKD2. PKD2-null ECs from these patients display aberrant localization of eNOS after the application of shear stress (1).

Recent *in vivo* studies of primary cilia complement previous *in vitro* studies of cilia. In zebrafish embryos, a morpholino against *Pkd2* blocks calcium influx in the arterial endothelium (52). *Tnnt2* and *gata1* morpholinos are also used, blocking heartbeat and reducing hematocrit, respectively. *Tnnt2* morphants display no ciliary deflection, while cilia in *gata1* morphants only deflect moderately. Importantly, endothelial calcium influx correlates with degree of ciliary deflection, as tnnt2 morphants show a severe reduction in EC calcium influx and gata1 morphants display only a moderate reduction (52, 101).

Despite the published work on the involvement of cilia in Ca^2+^ and NO signaling, the status of primary cilia as shear sensors remains unclear. Cilia reside in low flow areas of the vasculature (84, 87, 140, 144) and are mostly absent in areas of high flow. This suggests the intriguing possibility that cilia formation itself is a process that depends on blood flow, since cilia form as a response to low or disturbed flow. Although this does not exclude the possibility of cilia functioning as mechanosensors, their role as amplifiers of shear signals (32, 67, 81) is certainly compatible with their presence in areas of low flow. Cilia may act as low flow sensors in the endothelium, but disentangling ciliary functions in development and overall EC shear signaling remains a challenge.

### Glycocalyx

The endothelial glycocalyx consists of a mixture of glycoproteins, hyaluronin, and proteoglycans such as syndecan-1 and −4. The glycocalyx has a net negative charge due to the presence of glycoprotein-bound sialic acid moieties and proteoglycan-bound glycosaminoglycans (GAGs). As a result, an extended endothelial surface layer (ESL) is formed as a result of glycocalyx interaction with components of blood. The ESL comprises plasma proteins, growth factors, and cytokines (17, 111, 151).

Early work on the endothelial glycocalyx revealed a hydrodynamically relevant surface layer capable of regulating capillary hematocrit (35, 90, 116). Using *in vivo* solute exclusion assays, the height of the glycocalyx was determined to be approximately 500 nm. High molecular weight dextran was excluded from this 500 nm thick later, and the thickness of the layer was reduced by degrading the glycocalyx with an infusion of hyaluronidase (65, 145, 161). In addition, vascular TNFα infusion reduces glycocalyx height, suggesting that endothelial inflammation negatively regulates glycocalyx density (42, 66, 99). Degradation of hyaluronin or inhibition of its synthesis decreases glycocalyx thickness in collateral vessels, reducing collateral remodeling after hindlimb ischemia. Shear-dependent EC proliferation and inflammatory gene expression are also reduced (54, 72, 115).

The glycocalyx is thought to dampen the force of shear stress that reaches the EC surface (151). Thus, cells with an intact glycocalyx do not experience appreciable shear forces at their plasma membranes. Instead, syndecans link to the cytoskeleton through their cytoplasmic termini, providing a possible mechanism for force transfer from shear stress to the cytoskeleton (136, 143, 152). Indeed, ablation of syndecan function through enzymatic or genetic means leads to blockade of shear stress responses in ECs. Degradation of heparin sulfate GAGs, the major GAGs associated with syndecans, leads to misregulated actin dynamics in ECs. As a result, ECs do not break down their dense peripheral actin band and fail to form new stress fibers after application of shear (101, 135, 144, 151). Syndecan-1 knockout causes misregulation of RhoA and Akt, as well as upregulation of EC inflammatory gene expression after the application of shear stress (52, 146). Correlating with the syndecan-1 studies, syndecan-4 knockout ECs do not align in the direction of flow and display an increased inflammatory phenotype. This leads to a higher atherosclerotic burden *in vivo*. Importantly, in Syndecan-4 KD ECs, VEGFR2 phosphorylation is not altered immediately after application of shear. This indicates that syndecan-4-dependent signaling may act independently, or downstream, of junctional shear stress signaling (5, 67).

Enzymatic and genetic studies indicate the importance of glycocalyx components in shear-related signaling. However, syndecans act as coreceptors for integrins during adhesion, complicating the determination of specific function in shear stress signaling (64, 75, 95, 128, 136). Many EC responses that are misregulated in response to syndecan ablation, including RhoA activation and ICAM-1 and VCAM-1 expression, are downstream of integrin activation after the application of shear (87, 101, 140). Thus, syndecan function in shear signaling may be required for integrin function and not direct shear sensing. Further studies are required to completely disentangle specific glycocalyx signaling from integrin-associated signaling in ECs. The glycocalyx as an EC mechanosensor remains attractive, but clarity about its function remains elusive.

### Heterotrimeric G-proteins and G-protein-coupled receptors

Heterotrimeric G-proteins participate in shear stress signaling and may also form mechanosensitive complexes with known mechanosensors. Work done using vesicles loaded with G-proteins and ^32^P suggests that increasing membrane fluidity or applying shear stress leads to GTPase activation (56, 75). The heterotrimeric G-protein Gαq/11 associates with platelet endothelial cell adhesion molecule-1 (PECAM-1) in unstimulated ECs, rapidly dissociating upon application of shear. *In vivo,* Gαq/11 colocalizes with PECAM-1 in areas of laminar, but not disturbed, flow (34, 107). Interestingly, the association of Gαq/11 with PECAM-1 is mediated by heparan sulfate, suggesting a link between three candidate mechanosensors: PECAM-1, Gαq/11, and the glycocalyx (99, 112). Gαq/11 is also required for activation of Ras by flow, implicating G-proteins in shear-induced MAPK signaling (55, 101). In addition, siRNA knockdown of Gαq/11 increases the time required for calcium influx after the onset of retrograde shear stress (1, 96).

GPCRs are also implicated as mechanosensitive molecules. The bradykinin B2 receptor undergoes a ligand-independent conformational change in response to shear stress or membrane fluidizing agents (1, 16). Furthermore, sphingosine-1 phosphate receptor 1 (S1P_1_) is required for signal transduction of many well-characterized shear-responsive pathways, such as ERK, AKT, and eNOS activation. In this study, small-molecule induction of S1P_1_ proteasomal degradation blocked phosphorylation of ERK and EC alignment after application of shear stress. Interestingly, blockage of ERK activation and EC alignment was rescued using endocytosis- and ligand binding-deficient mutants of S1P_1_, indicating that the involvement of S1P_1_ in shear signaling is ligand independent (77). Endothelial S1P_1_ is also required for angiogenesis in the mouse retina (52, 77). These studies suggest that GPCRs and G-proteins are heavily involved in shear-responsive mechanotransduction and may be mechanosensors in their own right.

### Ion channels

Hyperpolarization of ECs was one of the first identified EC responses to shear and is among the earliest EC responses to shear (100, 102). K^+^ ion channel activity is regulated by shear stress-induced deformation of ECs, rather than high flow rate over ECs (3). After initial hyperpolarization of ECs in response to shear, Ca^2+^ channels open and depolarize the EC (8). Shear-induced Ca^2+^ influx increases with increasing magnitude of shear stress (155). Interestingly, the activity of one channel, transient receptor potential channel 5 (TRPC5), is enhanced by S-Nitrosylation (158). Although it is not known how shear stress might regulate S-Nitrosylation of TRPC5, the the S-Nitrosylation is dependent on eNOS, a shear-responsive protein (158).

Several ion channels are putative shear stress sensors in ECs. The endothelial Kir2.1 K^+^ channel has been shown to be mechanosensitive, and when expressed in *Xenopus laevis* oocytes, the oocytes become sensitive to shear (69). In addition, transient receptor potential vanilloid 4 (TRPV4) has been proposed as a mechanosensor. TRPV4 is sensitive to multiple stimuli, including heat, phorbol esters, arachidonic acid, hypotonic shock, and shear stress (47, 148–150). Interestingly, TRPV4 also has demonstrated importance in the vasculature, where it regulates vasodilation in carotid arteries, rat mesenteric arteries, and arterioles (40, 63, 80, 97) Regulation of vasodilation by TRPV4 may be achieved through TRPV4-dependent NO production from ECs (13). TRPV4 also forms a heterotrimeric flow-sensitive complex with TRPP2 and TRPC1 (38). TRPV4, however, is activated by arachidonic acid, which is synthesized by phospholipase A2 (PLA2) in response to shear stress (113). If PLA2 activity is inhibited, intercellular calcium and vasodilation responses are blocked (80, 91). Thus, TRPV4 activation is downstream of enzymatic activity and may not be sensitive to the force of flow. However, arachidonic acid synthesis takes place on a slower time scale (113) than TRPV4-dependent calcium flux (38), suggesting that there may be a yet undiscovered mechanism behind activation of TRPV4 by shear stress.

Recent work has identified the mechanically activated cation channels Piezo1 and Piezo2 in neurons (30). Piezo1 and Piezo2 are implicated in touch sensation in mice and red blood cell volume (14, 118) Transfection into nonmechanically activated HEK293T cells confers sensitivity to mechanical stimulation as well as shear stress (30, 31, 83). HEK293T cells transfected with Piezo1 exhibit increasing calcium influx with increasing shear stress. Piezo1 does not share structural features with proteins and may represent an entirely new class of mechanically activated ion channel (31). Piezo1 knockout mice are embryonic lethal as a result of major vascular defects manifesting as early as E8.5 (117). The vascular tree fails to prune correctly in both whole-body and EC-specific Piezo1 knockouts, and ECs in haploinsufficient arteries do not align in the direction of flow (83). Treatment of ECs with Piezo1 siRNA also largely blocks flow-induced EC alignment (117). Although little is known about these channels at the moment, they hold tremendous potential for the study of EC shear stress signaling.

### Caveolae

Caveolae are flask-shaped invaginations present in the plasma membrane of various cell types. Caveolae are mostly found in cell types that receive mechanical stress, such as fibroblasts, adipocytes, and ECs. Caveolae are given their structure by proteins of the caveolin and cavin family (CAV1-3 and cavin1-3, respectively) (41). Importantly, caveolae rapidly disassemble after hypotonic shock, suggesting a role in buffering the cell membrane against mechanical stress (127). Caveolae are also important in murine development, as Cav1 knockout mice display cardiac hypertrophy and thickened coronary vessel walls, along with bronchiolar hyperplasia (98).

Evidence for caveolae in endothelial mechanosensing was first described in rat lungs, where increased perfusion of the rat pulmonary vasculature led to enhanced protein tyrosine phosphorylation, especially in caveolar compartments (120). In addition, eNOS was enriched in rat lung EC caveolae, which served as the main location of eNOS activation after shear (119). The cholesterol binding antibiotic filipin and cholesterol sequestering compound methyl-β-cyclodextrin reduced shear-induced ERK activation in bovine aortic endothelial cells, suggesting that cholesterol-rich regions of the plasma membrane are required for shear signaling. However, a limitation of these pharmacological treatments is that both caveolae and lipid raft function are disrupted, complicating interpretation of results (110). Transmission electron microscopy images revealed a luminal surface increase in caveolae density in ECs subjected to shear stress (11). In addition, shear stress induced Cav1 translocation to the upstream edge of ECs, where hydrostatic pressure is highest on ECs (130). This correlates with the hypothesis that caveolae buffer cell membranes against high mechanical stress (127).

Genetic evidence for caveolae as mechanosensors has revealed a role for caveolae in transduction of shear stress signals, but not directly in mechanosensing. In one study, Cav1 knockout mice were subjected to partial carotid artery ligation. While WT mice showed a decrease in lumen diameter, the Cav1 knockout mice displayed no such change. However, the arterial walls of the Cav1 knockout carotids did thicken significantly, a result of significant increases in endothelial, medial, and adventitial proliferation (160). Furthermore, Cav1 knockout carotid arteries were deficient in vasodilation as a result of impaired eNOS activation (160). In a model of atherosclerosis, Cav1 whole body knockout mice were crossed to ApoE knockout mice, leading to a reduction in aortic plaque burden. The reduction in plaque burden caused by Cav1 knockout was accompanied by reductions in ICAM-1 and VCAM-1 expression (45). Interestingly, reconstitution of only endothelial Cav1 increased the aortic plaque burden and ICAM-1 and VCAM-1 expression back to control levels, suggesting that caveolin proteins mediate endothelial inflammation leading to atherosclerosis. Caveolae have also been linked to the actin cytoskeleton. Knockdown of Cav1 increases basal RhoA activity, blocking stress fiber formation in ECs (156). In addition, Cav1 is required for luminal β1 integrin activation after the onset of shear stress. This activation of luminal integrins does not require the actin cytoskeleton. When blocked, luminal β1 integrin activation reduces shear-induced Src family kinase, Akt, and eNOS signaling in ECs (157). Whether caveolae are mechanosensors is unclear and will take more integrative research to fully determine. Given that the caveolae number increases as a response to shear stress, it is likely that they do not act as sensors, but are rather shear regulated; however, more research could uncover a role for these structures in endothelial force sensing.

### Tie receptors

Members of the Tie family of receptor tyrosine kinases have also been implicated in shear stress sensing (20, 114). Tie1, a tyrosine kinase with immunoglobulin-like and EGF-like domains 1, is expressed in ECs and its expression maps distinctively to regions exposed to disturbed shear stress (154). *In vitro*, its expression is downregulated by laminar shear stress, while disturbed flow increases Tie1 promoter activity. Deletion of Tie1 increased activation of eNOS and resulted in decreased inflammatory signals in response to laminar flow *in vitro*. The same group demonstrated a dose-dependent reduction in atherosclerosis in Tie1-attenuated ApoE^−/−^ mice, giving credence to the idea that Tie1 is a critical regulator of the endothelial response to disturbed shear stress. Although it is mechanoresponsive insofar as it alters its expression and modulates downstream signaling, it is unclear if it is a direct sensor of shear stress.

## Junctional Mechanosensors

### Platelet endothelial cell adhesion molecule-1

**FIG. 4.**
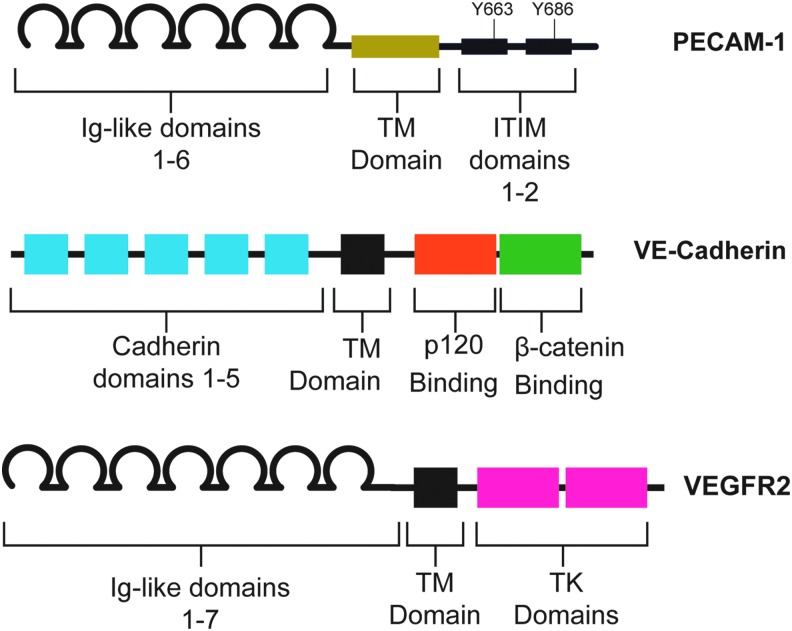
**Domain structure of the junctional mechanosensors.** The extracellular domain of PECAM-1 comprises six Ig-like repeats, which participate in homophilic binding with other PECAM-1 molecules on neighboring ECs. These Ig-like repeats are linked by a TM domain to the intracellular domain. The intracellular domain contains two ITIM domains, which contain critical tyrosines that are rapidly phosphorylated at the onset of shear. VE-Cadherin, a classical cadherin, has an extracellular domain that comprised five cadherin domains, which mediate adhesion, and features p120 and β-catenin binding sites on its intracellular domain. These intracellular domains interact with p120 and β-catenin, mediating VE-Cadherin binding to the cytoskeleton. VEGFR2, a receptor tyrosine kinase has seven Ig-like repeats in its extracellular domain. The intracellular domain contains two TK domains that interact with downstream effectors. Critically, tyrosines 801 and 1175 bind PI3K upon stimulation of the receptor. Mutation of these tyrosines blunts the cellular response to shear. Ig, immunoglobulin; ITIM, immunoreceptor tyrosine-based inhibitory motif; TK, tyrosine kinase; TM, transmembrane. To see this illustration in color, the reader is referred to the web version of this article at www.liebertpub.com/ars

PECAM-1 is an adhesion molecule of the immunoglobulin superfamily. The extracellular domain of PECAM-1, which mediates hemophilic binding to neighboring ECs, consists of six Ig-like subunits (Fig. 4). The intracellular and extracellular domains of PECAM-1 are linked by a short transmembrane domain. The major features of the intracellular domain are two immunoreceptor tyrosine-based inhibitory motif domains. These domains contain two tyrosines, 663 and 686, which are phosphorylated rapidly after the onset of shear stress. Along with Ca^2+^ influx and K^+^ channel activation, PECAM-1 phosphorylation is one of the earliest known responses to shear stress. Importantly, it is thought that PECAM-1 phosphorylation is independent of calcium influx, as Ca^2+^ agonists do not increase PECAM-1 phosphorylation on their own.

*In vitro* studies of the role of PECAM-1 in shear stress signaling are numerous. Early evidence implicating PECAM-1 in EC force sensing showed that PECAM-1 phosphorylation, Shp2 and Gab1 recruitment, and ERK phosphorylation all increase in response to hypo-osmotic shock and fluid shear stress (106). In addition, Shp2 phosphatase activity is required for PECAM-1-dependent ERK activation. Using an extracted EC model, Chiu *et al.* identified Fyn as the kinase that mediates stretch- and shear stress-induced PECAM-1 phosphorylation (22). Later evidence demonstrated that PECAM-1 acts in concert with VE-Cadherin and VEGFR2 to mediate a large number of shear stress responses. These responses include EC alignment, NF-kB activation, and Akt phosphorylation following application of shear stress (46, 141). Importantly, PECAM-1 is required for both anti-inflammatory and inflammatory signaling in ECs (141). PECAM-1 is required for establishment of polarity in ECs after onset of shear stress *via* spatiotemporal activation of Rac GTPase (86). In this model, PECAM-1 is required for Rac activation downstream of Src and Vav2 phosphorylation, while VE-Cadherin acts as a scaffold for localized Rac activation on the downstream edge of ECs. PECAM-1 knockout also leads to misregulation of eNOS localization and resultant increased NO production in unstimulated ECs (94). In addition, in PECAM-1 knockout ECs, eNOS activation is decreased in response to shear stress (46).

**FIG. 5.**
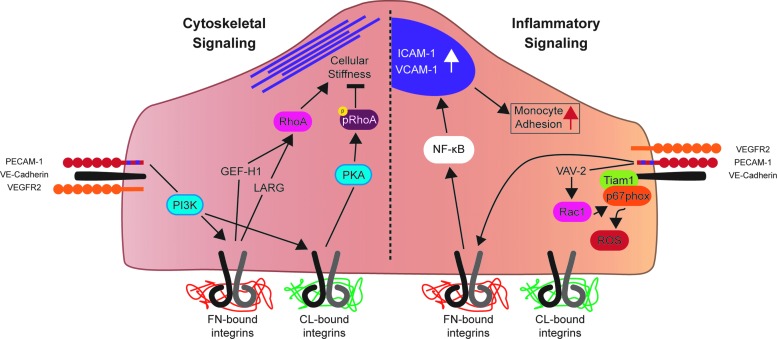
**The junctional mechanosensory complex regulates cytoskeletal stiffening and inflammatory signaling in response to force.** Force on PECAM-1 activates PI3K, which is required for downstream activation of integrins. ECM-dependent integrin activation occurs, with FN bound integrins positively regulating cellular stiffness through GEF-H1/LARG-RhoA signaling. CL bound integrins have the opposite affect; cellular stiffness is negatively regulated downstream of CL-bound integrins through a PKA-phospho-RhoA pathway. In addition, Rac1 is activated downstream of force on PECAM-1, which leads to increased inflamatory ROS production. In addition, shear stress activates FN-bound integrins, leading to NF-κB activation, ICAM-1 and VCAM-1 expression, and an increase in moncyte adhesion to “activated” ECs. CL, collagen; FN, fibronectin; PI3K, phosphoinositide 3-kinase; PKA, protein kinase A. To see this illustration in color, the reader is referred to the web version of this article at www.liebertpub.com/ars

Force application directly on PECAM-1 using ferromagnetic beads has allowed the study of specific PECAM-1-dependent signaling pathways (Fig. 5). Using magnetic beads coated with PECAM-1 antibody, early studies demonstrated that direct force on PECAM-1 elicits both PECAM-1 and ERK phosphorylation (106). PECAM-1 engagement is required for these responses, highlighting the importance of confluency in studies of EC shear stress signaling. Further studies of direct force application on PECAM-1 have shown that localized force on PECAM-1 can activate global cellular stiffening pathways in ECs. Application of force on PECAM-1 using magnetic beads manipulates only a small area of the EC; however, after 5 min of force, global focal adhesion growth and RhoA activation are observed (25). The observed focal adhesion growth and RhoA signaling culminate in cellular stiffening in response to force application. Interestingly, cellular stiffening, related cytoskeletal dynamics, and focal adhesion growth are all ECM-specific events. Plating ECs on collagen blocks force-induced focal adhesion growth and cytoskeletal dynamics that are normally observed in ECs growing on fibronectin (26). This suggests that PECAM-1 and integrins act cooperatively to mediate downstream signaling to force-based events. Since pharmacological inhibition of PI3-Kinase blocks force-induced focal adhesion formation and integrin activation (25), PECAM-1 may communicate with integrins *via* biochemical means, rather than direct force transfer through the cytoskeleton.

PECAM-1 null mice are healthy and fertile (39), which is surprising given the role of shear stress signaling in the development of the vascular plexus. However, starting at 4 weeks of age, PECAM-1 knockout mice display cardiac defects, including increased left ventricle diameter, reduced fractional shortening, and reduced ejection fraction (93). Flow-dependent dilation is blocked in *ex vivo* arteries from PECAM KO mice due to failure to activate eNOS (7). In addition, PECAM-1 KO mice subjected to partial carotid artery ligation display defects in flow-mediated vascular remodeling and intima-media thickening, due to defects in the NF-kB pathway (19). PECAM-1 KO mice also show reduced collateral remodeling after femoral artery ligation, once again due to reduced NF-kB-dependent transcriptional activity (18). Results from studies of atherosclerosis using PECAM-1 knockout mice have been difficult to interpret, possibly owing to differences in atherogenic genetic background and feeding time. However, across several studies, PECAM-1 knockout mice show reduced atherosclerotic plaque formation in the lesser curvature of the aortic arch (51, 61, 62, 129). This area is constantly exposed to disturbed flow, suggesting that PECAM-1 knockout dampens EC inflammatory signaling arising from disturbed flow.

### VE-Cadherin

We have previously shown that PECAM-1, VE-Cadherin, and VEGFR2 are sufficient and required for shear stress signaling in ECs (141). When VE-Cadherin-expressing cells are mixed with VE-Cadherin knockout cells and exposed to flow, the VE-Cadherin-expressing cells align in the direction of flow, whereas VE-Cadherin knockout cells do not (141). Thus, even in the absence of homophilic VE-Cadherin adhesion, ECs align correctly. This suggests that VE-Cadherin acts as an adaptor in shear stress signaling. VE-Cadherin knockout additionally blocks integrin activation after application of shear stress, but not Src activation (141). Src activation is one of the earliest events in shear stress signaling, taking place just after PECAM-1 phoshphorylation. This suggests that VE-Cadherin acts downstream of PECAM-1 in shear stress signaling, which allows transactivation of VEGFR2 and induction of downstream signaling pathways (87, 141). The VE-Cadherin transmembrane domain is also required for recruitment of VEGFR2 to the mechanosensory complex, further implicating VE-Cadherin as an adaptor in this system (Fig. 5) (29).

Recent evidence suggests that VE-Cadherin may also be a mechanosensitive molecule. Previous studies suggest that force application on VE-Cadherin using antibody-coated magnetic beads does not activate known force-sensitive signaling; however, recent evidence shows that this phenomenon may be epitope specific (9, 141). Application of force to VE-Cadherin using the VE-Cadherin FC antibody elicited responses similar to those elicited by force on PECAM-1; namely, cell stiffness and actin reorganization (9). In addition, FRET studies using PECAM-1 and VE-Cadherin tension sensors suggest that VE-Cadherin carries a high-tension load in unstimulated ECs. This load is reduced once shear stress is applied. Interestingly, tension on PECAM-1 is very low before application of shear stress. After the onset of shear, tension increases on PECAM-1 as a result of association with vimentin (28). The main role of VE-Cadherin in shear stress signaling is likely as an adaptor. However, the recent studies mentioned above imply that VE-Cadherin may have other roles in EC shear stress signaling. More research is needed to determine whether the force-sensing properties of VE-Cadherin are relevant in physiological signaling.

### VEGF receptors

VEGF receptors 2 and 3 (VEGFR2 and VEGFR3) are also involved in shear stress sensing. VEGFR2, the better characterized of the two, is rapidly phosphorylated at the onset of shear (87, 125, 141). Shear stress-induced Akt activation is blocked when tyrosines 801 and 1175 of VEGFR2 are mutated, implying that VEGFR2 is required for shear-induced PI3 kinase activation and resultant downstream signals (Fig. 5). Thus, while there is no evidence to suggest VEGFR2 is directly mechanosensitive, its activity is required for intact shear stress signaling.

Recently, VEGFR3 was shown to be as a constituent of the mechanosensory complex containing PECAM-1, VE-Cadherin, and VEGFR2. This evidence also implicates VEGFR3 in sensing different magnitudes of shear, adding to our understanding of how different shear patterns are sensed. At the onset of shear, VEGFR2 and VEGFR3 are recruited to the mechanosensory complex by the transmembrane domain of VE-Cadherin, followed by phosphorylation of both VEGFR2 and VEGFR3 (29). Evidence also suggests that VEGFR2 and VEGFR3 signal redundantly in response to shear stress. Knockdown of both proteins, but not VEGFR2 or VEGFR3 individually, blocks shear-induced Akt activation (29). Interestingly, VEGFR3 has been implicated in determining the sensitivity of different types of ECs to shear stress (6). Human dermal lymphatic endothelial cells (HDLECs) and HUVECs, when exposed to a range of shear stress levels, show peak alignment at different shear magnitudes. HDLECs align most efficiently around 5 dyn/cm^2^, while HUVECs align most efficiently around 10 dyn/cm^2^. In addition, HDLECs express higher levels of VEGFR3 than HUVECs do. HDLECs phenocopy HUVECs upon knockdown of VEGFR3, aligning at higher levels of shear than normal HDLECs. Taken together, these results suggest that shear stress sensing may be more variable across the phenotypic spectrum of ECs than previously thought. Endothelial mechanosensing may not simply be a matter of efficient force transmission and biochemical information transfer by one or more specialized EC proteins, the mechanisms of which are uniform across ECs. Mechanisms of mechanosensing may vary with endothelial niche bestowed upon them by differential expression of mechanosensory constituents.

## Basal sensors: Integrins

Integrins act as connections between the ECM and the actin cytoskeleton, making them attractive candidate mechanosensors in ECs. However, evidence that integrins are direct shear stress sensors in ECs is limited. After application of shear stress or force on PECAM-1, integrins are activated globally in ECs (25, 140, 141). This global activation requires PI3 kinase, suggesting that shear-induced activation of integrins is mediated by a biochemical stimulus, such as PIP_3_, rather than force transfer through the cytoskeleton (25, 141). In addition, the amount of force that integrins feel from shear stress is 1000–5000 times lower than traction forces on integrins (79). This further suggests that integrins are not activated by the direct force of shear stress. However, it has been hypothesized that mechanosensitive cells have evolved specialized mechanosensors capable of sensing extremely small forces unique to their niche (103). Thus, it is possible that the integrins act as specialized endothelial mechanosensors. Integrins are mechanosensitive proteins and, as such, could be responding to minute shear stress forces or changes in cytoskeletal tension (23, 57, 92, 147) (For further information about the interaction between hemodynamics, integrins, and the ECM, please see Yurdagul and Orr later in this Forum).

Integrins serve as the integration site for shear-dependent signaling cascades (87, 139, 140). After the application of shear on ECs, integrins are activated and form new connections with the underlying ECM. These new connections with the ECM are required for downstream activation of RhoGTPases, and NF-κB (139, 140, 142). Integrins also influence the endothelial phenotype through integration of upstream signals with signals from the ECM. The adaptor protein Shc, which binds with the mechanosensory complex at the onset of shear, binds integrins around 30 min after the onset of shear (76, 87). As a result of interaction with two separate mechanosensors, Shc integrates signals of junctional and subendothelial origin in both shear and nonshear contexts (87, 132, 133). In addition, focal adhesion growth resulting from force on PECAM-1 requires fibronectin; no growth is observed when cells are plated on collagen (26). Collagen binding also regulates cytoskeletal dynamics in ECs. When force was applied directly to PECAM-1 in ECs grown on collagen, protein kinase A (PKA) was activated, leading to repression of RhoA activity and reduction of cell stiffness, while the opposite was true of ECs grown on fibronectin (26). Passive rheology experiments performed on isolated mouse aortas confirmed these observations. EC stiffness was high in the aortic arch, where fibronectin density is high, while EC stiffness was lower in the descending aorta, where collagen makes up most of the ECM. Interestingly, *in vivo* inhibition of PKA reversed this effect, causing higher EC stiffness in the descending aorta as a result of removal of RhoA repression (26). In addition, ECs plated on collagen align less effectively than cells plated on fibronectin (59). Other shear-induced signaling pathways are also matrix specific. Shear-induced phosphorylation of p21-activated kinase (PAK) requires fibronectin connections, while shear-induced phosphorylation of p38 MAP kinase occurs only on collagen (104, 105). Furthermore, disturbed flow regimens induce fibronectin expression and deposition, which sustain endothelial inflammation through NF-kB-dependent gene expression (44). Although integrins likely do not directly sense shear stress, their function as integrators of shear stress signaling pathways demonstrates that they are an integral part of the EC shear sensing milieu.

## Conclusions

### What is a mechanosensor? Do they work alone?

A mechanosensor is the primary cellular structure or protein that senses a physical force, setting off downstream biochemical signaling pathways. ECs may have one, but their number is likely many. PECAM-1, the best studied of the putative EC mechanosensors, is capable of initiating global biochemical/signaling cascades in the cell when force is applied on it. A mechanotransducer is a cell structure or protein that is required for conductance of signals generated by force, but not for sensing the originating force itself. In ECs under shear stress, integrins likely fall into this category as their activation seems to originate from biochemical and not force-based signals in ECs. The status of the other putative mechanosensors mentioned in this article remains unclear, however. Further experiments must be done to eliminate the caveats inherent in the knowledge of each mechanosensor. Only then can we definitely assign force-sensing properties to putative EC mechanosensors and begin untangling the signaling relationships they share.

An unanswered question is the extent to which the putative mechanosensors work together to sense flow and maintain EC mechanosensitivity. There may be only one ultimate mechanosensor in ECs, but this seems unlikely given the known cooperation between sensors. Perhaps the best example of this is the cooperation between PECAM-1 and integrins, but evidence is starting to accumulate that other mechanosensors cooperate as well. For example, it has been shown that ligand-independent S1P_1_ activity is required for maintenance of the glycocalyx, a possible example of mechanosensor cooperation as well as a possible mechanism for specific localization of the glycocalyx in areas of laminar flow (162). In addition, the heterotrimeric G-protein Gαq/11 complexes with PECAM-1, suggesting that more than one mechanosensor may be working together to sense flow (112). Interestingly, the Gαq/11–PECAM-1 interaction is mediated by heparin sulfate, a component of the glycocalyx, further suggesting mechanosensor cooperation. Finally, one of the roles of primary cilia is thought to be amplification of signals through the cytoskeleton. This could be an instance where cilia act alongside other major mechanosensors to help sense the smaller forces from low flow and influence EC phenotype. Related questions center around the relationship between shear stress and cyclic stretch. Although some *in vitro* studies (163) suggest that stretch and shear signaling are interrelated processes, little is known about any overlap in these two signaling cascades. To understand the full scope of vascular mechanosignaling, knowledge of the mechanisms of cyclic stretch signaling and shear signaling must be incorporated.

### Developmental questions and endothelial heterogeneity

Mice with deleted PECAM-1, Syndecan-1, and Syndecan-4 are healthy and fertile, although PECAM-1 knockouts suffer from dilated cardiomyopathy and systolic dysfunction (93). In knockout mice for other mechanosensory genes, such as *Pkd1* or *Pkd2*, defects in development have not been disentangled from any possible defects in shear stress signaling (2, 5, 48). A major issue for the shear stress field moving forward is understanding the importance of shear sensing *in utero* and the signaling pathways involved. Similarly, another challenge for the field is understanding the phenotypes of ECs from different vascular beds and how those phenotypes influence shear signaling. Already we have evidence that a shear sensing set point may be regulated by VEGFR3 expression in lymphatic ECs (6). This suggests the tantalizing possibility that specialized vascular beds, such as lymphatic, brain, or renal ECs, may be specialized in their function and methods of shear sensing. Perhaps, for different vascular beds, one mechanosensor does not fit all.

### Clinical perspectives

Shear stress and therefore endothelial mechanosignaling are central to many vascular pathologies. Atherogenesis, for example, preferentially occurs in areas of the vasculature that are inflamed due to experiencing disturbed flow (17). Furthermore, shear stress may influence the rupture of unstable plaques. The fibrous cap of a plaque is exposed to the highest strain, and shear stress, at its upstream edge (49). Previous work has shown that macrophage invasion is higher in the upstream edge of plaques (24, 36) and that resident macrophages in the upstream edge increase expression of proteolytic enzymes (24), thereby weakening the plaque. In addition, the fibrous cap on the upstream edge of plaques tends to be more ulcerated and thinner and, when combined with high shear stresses, may lead to destabilization and rupture of the plaque (33, 49). Another common vascular pathology, intimal stiffening, occurs with age. Intimal stiffening is associated with increased RhoA activity and vascular permeability, leading to increased leukocyte transmigration (74). In addition, conditions that affect vasodilation, such as aging and hypertension, modify the hemodynamic environment. In tests of femoral artery shear rates in old and young men, old men showed lower shear rates (137), predisposing the older men to endothelial inflammation. Flow patterns are also altered in older men: anterograde flow is reduced, while retrograde flow is increased. These changes combine to increase the overall oscillatory nature of blood flow in the femoral artery, once again predisposing older men to increased endothelial inflammation (159). While exercise transiently reduces age-related increases in oscillatory shear index (108), much work remains before the full involvement of endothelial mechanosignaling in a range of vascular pathologies can be determined.

### Future directions

**FIG. 6.**
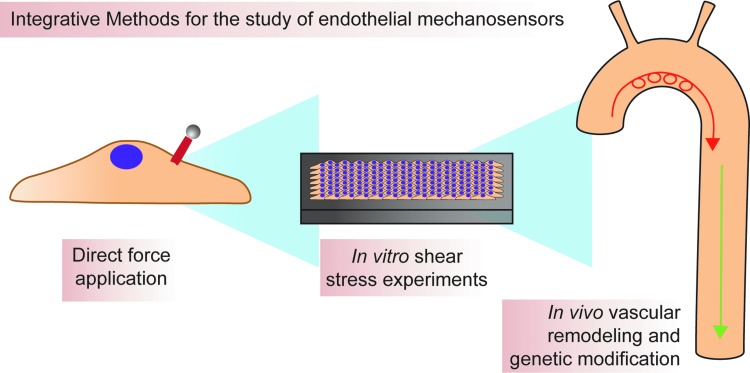
**Integration of approaches is necessary for determining the precise function of various mechanosensors.** Methods available for the study of mechanosensing range from reductionist biophysical experiments to *in vivo* hemodynamic modification. Magnetic beads are used to apply force directly to putative mechanosensors and can be useful for directly determing mechanosensitivity of a protein. *In vitro* shear stress experiments are useful for determining mechanoresponsiveness of proteins in the context of an endothelial monolayer, while *in vivo* experiments determine whether information gained using the aforementioned reductionist approaches is physiologically relevant. To see this illustration in color, the reader is referred to the web version of this article at www.liebertpub.com/ars

How should new mechanosensors be discovered? How should putative mechanosensors be validated? There are many *in vitro* and *in vivo* models for determining function of cellular structures or proteins in shear sensing (for more in depth information on these models, please see Bowden *et al.*, later in this issue). Moving forward, every effort should be made to mix reductionist and integrative approaches as much as possible (Fig. 6). For example, magnetic bead pulling experiments are an excellent way to assign force-sensing function to a protein. However, these experiments do little to illuminate how that protein may behave in the context of a monolayer or an adult animal. Likewise, simply studying mouse knockouts or *in vitro* models of shear makes assignment of direct force-sending function problematic. By mixing reductionist and integrative approaches, we can better understand known putative mechanosensors and more easily find new candidate mechanosensors. When the mechanosensory infrastructure of ECs is more fully understood, a more full knowledge of intracellular signaling pathways will follow. By studying these pathways using a mixture of *in vitro* and *in vivo* approaches, the aim is that one or more signaling pathways that regulate inflammatory, but not atheroprotective, signaling are identified. Discovery of such a pathway could allow rational design of treatments that block endothelial inflammation, and thus, development of atherosclerosis and other shear-induced vascular pathologies. This could lead to novel therapeutics of enormous potential.

## Abbreviations Used

CL: collagen
EC: endothelial cell
ECM: extracellular matrix
ESL: endothelial surface layer
FN: fibronectin
GAG: glycosaminoglycans
GPCR: G-protein-coupled receptor
HDLEC: human dermal lymphatic endothelial cell
HUVEC: human umbilical vein endothelial cell
Ig: immunoglobulin
ITIM: immunoreceptor tyrosine-based inhibitory motif
NO: nitric oxide
PECAM-1: platelet endothelial cell adhesion molecule-1
PI3K: phosphoinositide 3-kinase
PKA: protein kinase A
PLA2: phospholipase A2
ROS: reactive oxygen species
S1P_1_: sphingosine-1 phosphate receptor 1
TK: tyrosine kinase
TM: transmembrane
TRPC5: transient receptor potential channel 5
TRPV4: transient receptor potential vanilloid 4
